# Gene Expression Analysis of a Panel of Cell Lines That Differentially Restrict HIV-1 CA Mutants Infection in a Cyclophilin A-Dependent Manner

**DOI:** 10.1371/journal.pone.0092724

**Published:** 2014-03-24

**Authors:** Vaibhav B. Shah, Christopher Aiken

**Affiliations:** Department of Pathology, Microbiology and Immunology, Vanderbilt University School of Medicine, Nashville, Tennessee, United States of America; Meharry Medical College, United States of America

## Abstract

HIV-1 replication is dependent on binding of the viral capsid to the host protein cyclophilin A (CypA). Interference with cyclophilin A binding, either by mutations in the HIV-1 capsid protein (CA) or by the drug cyclosporine A (CsA), inhibits HIV-1 replication in cell culture. Resistance to CsA is conferred by A92E or G94D substitutions in CA. The mutant viruses are also dependent on CsA for their replication. Interestingly, infection of some cell lines by these mutants is enhanced by CsA, while infection of others is not affected by the drug. The cells are thus termed nonpermissive and permissive, respectively, for infection by CsA-dependent mutants. The mechanistic basis for the cell type dependence is not well understood, but has been hypothesized to result from a dominant-acting host factor that blocks HIV-1 infection by a mechanism that requires CypA binding to the viral capsid. In an effort to identify a CypA-dependent host restriction factor, we adopted a strategy involving comparative gene expression analysis in three permissive and three non-permissive cell types. We ranked the genes based on their relative overexpression in non-permissive cell types compared to the permissive cell types. Based on specific selection criteria, 26 candidate genes were selected and targeted using siRNA in nonpermissive (HeLa) cells. Depletion of none of the selected candidate genes led to the reversal of CsA-dependent phenotype of the A92E mutant. Our data suggest that none of the 26 genes tested is responsible for the dependence of the A92E mutant on CsA. Our study provides gene expression data that may be useful for future efforts to identify the putative CypA-dependent HIV-1 restriction factor and in studies of other cell-specific phenotypes.

## Introduction

Cyclophilin A (CypA) is a cellular peptidyl-prolyl isomerase that interacts with the HIV-1 capsid and is important for productive infection by the virus [1]–[5]. Interaction of CypA with the viral Gag polyprotein in the producer cells leads to incorporation of CypA in the budding virions [5], yet it is the interaction of CypA with the incoming viral capsid in the target cell that appears to account for the role of CypA in HIV-1 infection [6], [7]. CypA binds to an exposed loop on the surface of the CA protein [2]. The CypA-binding loop consists of Pro85 to Pro93, with Gly89 and Pro90 constituting the binding site for CypA [2]. Preventing the CA-CypA interaction by the immunosuppressive drug cyclosporine A, which targets all cyclophilins [8], or by mutating CypA-binding residues in CA leads to impaired infectivity in most cell types [1], [5]–[7], [9]–[14]. The effect of CypA appears to occur during a post-entry step of the virus life cycle subsequent to reverse transcription [12]–[14]. However, the exact mechanism by which CypA promotes HIV-1 replication remains unknown.

Inhibition of the CypA-CA interaction in HeLa cells by CsA, or its analogs leads to impaired HIV-1 replication [15]–[17]. However, these inhibitors have minimal effect on the early post- entry steps of the virus life cycle in HeLa cells [6], [7], [18], [19]. Passage of HIV-1 in HeLa-CD4^+^ cells in the presence of CsA led to selection of CsA-resistant CA mutants A92E and G94D [15], [20]. These substitutions do not detectably alter CypA-CA binding interactions [20]. Additional mutants exhibiting CsA resistance have also been identified outside the CypA binding loop [21]–[23]. Interestingly, in some cell types replication of the CsA-resistant mutants requires CsA or depletion of CypA, indicating that CypA inhibits infection in these cells [6], [7], [24]. The mechanistic basis for this cell-specific restriction is not well understood. The cell-type specific nature of the CsA-dependent mutants suggests the presence of an inhibitory factor in non-permissive cells. Alternatively, a host factor might facilitate infection of the permissive cell lines by the mutants. To distinguish between these two possibilities, Song and Aiken [18] generated heterokaryons by fusion of permissive 293T cells and nonpermissive HeLa-P4 cells. Infection of the heterokaryons by the mutants was enhanced by addition of CsA, suggesting the presence of a dominant-acting, CypA-dependent restriction factor in cell lines not permissive to infection by the mutants.

A common characteristic of all the known CsA-dependent mutants is that they are rescued by a specific second-site suppressor mutation [13], [22], [23] suggesting marked phenotypic similarity, thus they can be grouped together as a single class. In the present study, we sought to identify a cell-specific host factor that restricts this class of mutants in a CypA-dependent manner, using A92E as a representative example. We quantified expression of genes in permissive and non-permissive cell lines using a human gene microarray and ranked them in order of fold-expression in non-permissive vs. permissive cell lines. We selected 26 genes with at least 3-fold higher expression in non-permissive cell lines, knocked them down individually in a non-permissive cell line, and analyzed the effects on CsA-dependent infection by HIV-1-A92E. We did not identify a gene responsible for reduced infectivity of A92E mutant in non-permissive cell lines. Nonetheless, the gene expression data reported here can be employed to guide future efforts to identify the putative CypA-dependent restriction factor as well as other host genes responsible for other cell-specific phenotypes.

## Methods and Materials

### Plasmids and Chemicals

A92E CA mutant was subcloned from HIV-1 proviral DNA construct R9 [25] by transfer of ApaI-SpeI or BssHII-SpeI restriction fragments into HIV-GFP [26], an envelope-defective pNL4-3-based HIV-1 reporter virus clone encoding green fluorescent protein (GFP) in place of Nef. The presence of the mutations in the final constructs was confirmed by DNA sequencing. Plasmid pHCMV-G encodes a vesicular stomatitis virus G (VSV-G) protein under the control of the human cytomegalovirus (CMV) promoter [27]. Cyclosporine (CsA) was purchased from Calbiochem and dissolved in ethanol to produce a 1 mM stock concentration.

### Cells and Viruses

Jurkat, CEM, H9 and HOS cells were obtained from NIH AIDS Research and Reference Reagent Program as contributed by Dr. Arthur Weiss, Dr. J.P.Jacobs, Dr. Robert Gallo and Dr. Nathaniel Landau, respectively [28]–[32]. HeLa and 293T cells were kind gifts by Dr. Eric Freed and Dr. Inder Verma respectively [33], [34]. HeLa, HOS and 293T cells were cultured in Dulbecco’s modified Eagle’s medium (DMEM) (Cellgro) supplemented with 10% fetal bovine serum (FBS), penicillin (50 IU/ml), and streptomycin (50 μg/ml) at 37°C with 5% CO_2_. Jurkat, CEM and H9 cells were cultured in RPMI media supplemented with 10% fetal bovine serum (FBS), penicillin (50 IU/ml), and streptomycin (50 μg/ml) at 37°C with 5% CO_2_. Virus stocks were produced by calcium phosphate transfection of 293T cells [35]. VSV-G-pseudotyped reporter virus particles were produced by cotransfection of 15 μg of wild-type (WT) or mutant HIV-GFP plasmid and 5 μg of pHCMV-G plasmid DNA. Two days after transfection, culture supernatants were harvested, clarified by filtration through 0.45-μm-pore-size filters, and frozen into aliquots at −80°C. The CA content of virus stocks was quantified by a p24-specific enzyme-linked immunosorbent assay (ELISA), as previously described [36].

### RNA Isolation for Microarray

Total RNA was isolated from ∼1×10^6^ HeLa-P4, HOS and 293T cells, and ∼5×10^6^ Jurkat, CEM and H9 cells seeded the day prior. RNA was purified with TRIZOL per the manufacturer’s protocol. RNA was further purified using RNeasy kit (Qiagen) followed by on-column DNaseI treatment. The RNA concentration was determined by spectrophotometry, and RNA integrity was analyzed by agarose gel electrophoresis.

### Genome-Wide Expression Analysis with the Human Gene 1.0 ST Array

In order to target each “gene” for the desired level of probe coverage on the Gene 1.0 ST Arrays the boundaries of each gene were defined by calculating “gene bounds”. Conceptually, gene bounds are the projection of all exons for a given gene onto the genome. The gene bounds were calculated in a hierarchical manner, using better-annotated evidence first (i.e., RefSeq) and more speculative content later (i.e., Ensembl predictions). For the Human Gene 1.0 ST Array, only RefSeq, Ensembl and putative complete CDS mRNA from GenBank were used to generate gene-bound annotations. To ensure robust gene-level estimates of expressed RNA, a target of 25 probes per gene was selected for each gene bound. Preference was given to probes already on the Human Exon 1.0 ST Array. In short, probes were selected uniformly over the gene structure. Some of the gene bounds were covered by less than 25 probes from the Human Exon 1.0 ST Array, so additional probes were selected from the genome sequence for the gene bounds. In these cases new probes were picked uniformly over the gene structure. Around 80 percent of the probes on the Human Gene 1.0 ST Array are also present on the Human Exon 1.0 ST Array. To prepare DNA for hybridization to the array, total cellular RNA was random-primed to generate double-stranded DNA using Affymetrix WT cDNA synthesis kit. After a clean-up step, double-stranded cDNA was fragmented and end-labeled with terminal deoxynucleotidyl transferase before hybridization overnight onto Vanderbilt Microarray Shared Resources (VMSR) Human Gene 1.0ST array. The single array contained 28,869 gene-level probe sets composed of 764,885 distinct probes, with a mean of 28 probes per gene and a median of 26 probes per gene. Various control probe sets such as hybridization control probe sets for BioB, BioC, BioD and CreX pre-labeled spikes, assay control probe sets for the Dap, Phe, Lys and Thr polyA unlabeled spikes, putative exon and intron control probe sets from putative constitutive genes for use as pseudo positive/negative controls and generic background probes were also included in the array. All data were RMA normalized using Partek.

### siRNA Knockdown and viral Infectivity Assay

HeLa-P4 cells were transfected with either SMARTpool siRNAs (Dharmacon) or scramble siRNA using siPORT NeoFX transfection reagent (Invitrogen) per the manufacturer’s protocol. As a control for functional knockdown, we transfected a parallel culture with a well-characterized siRNA against TNPO3, a protein on which HIV-1 infection is dependent. 72 hours after transfection, cells were trypsinized and replated in 24-well plates at a density of 25,000 cells/well. The next day, monolayers were inoculated with VSV-G-pseudotyped HIV-GFP-A92E in the presence or absence of CsA (5 μM). Twenty-four hours later, the cultures were supplemented with 1 ml of additional medium and incubated for another 24 h. Cells were then detached with trypsin, fixed in 2% paraformaldehyde, and analyzed for GFP expression with an Accuri C6 flow cytometer.

## Results

### Cell-type-dependent Phenotype of the A92E Mutant

Previously, several studies have demonstrated CsA-dependence of the A92E mutant in HeLa and H9 cells [6], [24]. In order to identify other cell types in which infection by the A92E mutant is dependent on CsA, we tested several cell lines for VSV-G-pseudotyped-A92E infectivity in the presence or absence of CsA. As shown previously [6], [24], we observed that CsA enhanced infection of HeLa and H9 cells 6.5-fold and 2.0-fold, respectively (Table 1). In addition to H9 cells, we employed another T cell line, CEM, in which CsA enhanced A92E infection by 1.8-fold (Table 1). In contrast to the non-permissive cell lines, we observed that CsA exhibited minimal effects on infection by the A92E mutant in HOS and 293T cells. Moreover, in Jurkat cells, CsA inhibited infection by approximately 50%.

**Table 1:** Phenotype of the A92E mutant in different cell lines.

Cell type	Experiment 1			Experiment 2			
	% GFP cells			% GFP cells			
	−CsA	+CsA	fold change (+CsA/−CsA)	−CsA	+CsA	fold change (+CsA/−CsA)	Mean fold change
HeLa	3.8	23.3	6.2	5.0	37.5	7.5	6.5
H9	3.2	7.8	2.5	8.3	12.0	1.4	2.0
CEM	11.4	21.3	1.9	8.8	15.5	1.8	1.8
Jurkat	13.0	5.9	0.5	26.4	11.7	0.4	0.4
HOS	15.7	19.0	1.2	17.2	22.0	1.3	1.2
293T	8.7	7.6	0.9	26.1	15.4	0.6	0.7

Indicated cell lines were inoculated with different amounts of HIV-GFP-A92E virus particles in the presence or absence of CsA (5 μM). Infectivity was quantified 48 hours after infection by flow cytometry. Shown are the infectivity values obtained with infection of H9, CEM, Jurkat and 293T cells by 2.5 ng p24 and of HeLa and HOS by 0.5 ng p24 of the mutant virus particles.

### Human Gene Array

To identify genes that may play a role in the CypA-dependent restriction of the A92E mutant, we performed Human Gene Array analysis of the six cell lines in triplicate. In all, expression of 28,869 genes was analyzed across these six cell types (complete data set shown in Table S1). The cell lines were categorized as nonpermissive (HeLa, H9, CEM) and permissive (Jurkat, HOS, 293T). For each gene, the mean expression in the permissive cell group was compared to the corresponding value for the non-permissive group. In this way, the fold-change in expression in non-permissive cells to permissive cells was determined for each of the 28,869 human genes. The genes were then sorted by the ratio of the expression in non-permissive cells versus permissive cells (Table S2). The top 100 candidate genes with at least 3-fold higher expression in non-permissive cells compared to permissive cells were selected. This list was further scrutinized based on two criteria: (1) Expression of a gene in HeLa cells should be higher than in any of the permissive cell types because A92E demonstrated the most pronounced CsA-dependent phenotype in HeLa cells; and (2) Expression of a gene in any of the permissive cell types should be lower than in at least two of the non-permissive cell types. Using these two criteria, 26 genes (Table 2) were selected for analysis of their effects on HIV-1-A92E infection.

**Table 2:** Genes selected from the microarray data for further analysis.

Gene	Expression (log2) (RMA* normalized)						fold expression#
	HeLa	H9	CEM	Jurkat	HOS	293T	(non-permissive/permissive)
TES	11.70	11.70	9.87	5.06	5.13	9.77	23.0
VAMP8	10.36	10.56	10.75	6.65	6.47	6.61	16.0
ACSL5	7.58	10.47	9.63	6.17	5.97	5.26	11.0
IFITM1	9.16	11.41	10.85	8.13	6.26	7.01	10.0
L1CAM	10.61	8.91	5.52	5.38	5.61	5.34	7.5
S100A11	12.45	11.51	11.06	5.70	11.78	8.82	7.5
SLC16A6	11.18	9.49	7.87	6.92	6.67	6.37	7.1
C6orf150	9.95	9.27	8.55	5.33	9.83	4.99	5.8
TNS4	7.35	11.26	5.96	5.75	5.83	5.58	5.6
JUP	10.55	11.13	6.91	7.04	6.74	7.73	5.2
AGMAT	8.96	7.74	8.72	6.03	6.06	6.49	4.9
STAT5A	8.58	7.90	10.52	7.67	6.30	6.20	4.9
PRSS21	10.36	8.62	10.10	7.45	7.68	7.16	4.8
CD44	11.29	11.80	9.12	6.29	12.54	6.62	4.8
MCTP2	9.30	7.17	4.80	4.64	5.45	4.47	4.7
SLCO3A1	11.16	10.02	7.57	6.57	8.16	7.30	4.7
MEST	9.92	10.22	5.90	6.14	7.86	5.49	4.6
BST2	10.07	8.24	9.53	8.97	6.18	6.19	4.6
CXCR4	10.60	11.65	11.28	12.25	7.99	6.80	4.5
S100A4	9.49	8.89	6.74	6.63	6.40	5.62	4.5
PLEKHG1	8.38	9.54	5.11	5.04	5.31	6.26	4.5
ICAM1	9.97	6.30	6.80	6.43	6.31	5.89	4.5
NEDD9	9.52	8.86	6.15	7.13	5.64	5.66	4.1
ITGB8	8.12	7.85	4.97	4.82	4.93	5.22	4.0
TLL1	9.02	7.20	5.13	5.08	5.18	5.30	3.8
MLH3	7.50	8.21	8.31	5.03	7.93	5.32	3.8

*Robust Multiarray Averaging.

# Fold-change in expression was calculated by converting the expression values to linear scale, averaging gene expression in each group and taking ratio of expression in non-permissive to permissive groups.

### Analysis of the Candidate Genes

To test if expression of any of the selected candidate genes is necessary for the observed CsA-dependent phenotype of the A92E mutant, each of these genes was individually knocked down in HeLa cells using SMARTpool siRNAs. For the assays, HeLa cells were chosen because the A92E mutant demonstrated the strongest enhancement by CsA in this cell type. A scrambled siRNA sequence was used as a control for non-specific effects of siRNA transfection. The analysis was performed in several experiments with subsets of the siRNAs, each of which also contained the scrambled control. Seventy two hours after siRNA transfection, cells were harvested and plated for infection. Cells were transduced with VSV-G-pseudotyped HIV-GFP-A92E mutant particles in the presence or absence of CsA sixteen hours after plating. Transfected cells were analyzed for single-cycle infection by the mutant forty-eight hours after inoculation. No apparent cytotoxicity was observed with knockdown of these candidate genes using 200 nM of their respective siRNAs. We reasoned that depletion of the restriction factor would result in enhancement of HIV-1-A92E infection in the absence of CsA, and would have minimal effects on infection in the presence of CsA, resulting in a net reduction in the extent to which CsA enhances infection of the virus. We observed that depletion of any of the 26 candidate genes did not result in complete rescue from CsA dependence (Table 3). However, depletion of nine genes including *TLL1*, *AGMAT*, *NEDD9*, *PRSS21*, *IFITM1*, *PLEKHG1*, *SLC16A6, ICAM1* and *ACSL5* resulted in 1.6–2.7-fold enhancement of A92E infectivity in the absence of CsA and 1.4–2.0-fold decrease in CsA enhancement of A92E infection. In addition, depletion of these 26 genes had no significant effect on infectivity of the wild-type virus (data not shown).

**Table 3:** Analysis of selected genes by siRNA knock down.

	% GFP +ve cells						fold change inCsA dependence	fold change in infectivityin the absence of CsA
	scramble		KD		scramble	KD		
	−CsA	+CsA	−CsA	+CsA	+CsA/−CSA	+CsA/−CSA	(scramble/KD)	(KD/scramble)
TLL1	1.6	12.1	4.2	16.3	7.8	3.9	2.0	2.7
AGMAT	1.9	14.7	4.9	23.6	7.9	4.8	1.6	2.6
NEDD9	1.6	10.8	4.0	16.8	6.8	4.3	1.6	2.5
PRSS21	1.9	14.7	3.8	19.0	7.9	5.1	1.6	2.0
IFITM1	2.2	14.2	3.7	15.9	6.6	4.3	1.5	1.7
PLEKHG1	1.6	10.8	3.3	14.3	6.8	4.4	1.5	2.0
SLC16A6	2.9	14.3	4.8	15.6	5.0	3.3	1.5	1.7
TES	2.2	14.2	2.8	12.7	6.6	4.5	1.5	1.3
ACSL5	2.9	14.3	5.3	18.7	5.0	3.5	1.4	1.9
VAMP8	1.9	14.7	2.5	14.0	7.9	5.7	1.4	1.3
ICAM1	1.6	10.8	3.1	15.3	6.8	4.9	1.4	1.9
L1CAM	2.9	14.3	4.8	17.8	5.0	3.7	1.3	1.7
Corf6	2.9	17.4	2.8	12.5	6.0	4.5	1.3	0.9
STAT5A	1.9	13.0	2.2	11.8	7.0	5.3	1.3	1.2
S100A11	2.9	17.4	4.1	18.6	6.0	4.6	1.3	1.4
MLH3	3.6	20.0	4.3	19.0	5.6	4.5	1.3	1.2
CXCR4	3.6	20.0	4.8	22.1	5.6	4.6	1.2	1.4
SLC03A1	1.0	8.5	1.3	8.9	8.5	7.1	1.2	1.3
JUP	1.9	13.0	2.0	11.9	7.0	5.9	1.2	1.1
BST2	2.2	14.2	3.2	17.9	6.6	5.6	1.2	1.5
MEST	1.0	8.5	1.7	12.6	8.5	7.4	1.1	1.7
S100A4	1.6	10.8	2.0	12.1	6.8	6.0	1.1	1.3
TNS4	1.9	13.0	2.4	15.4	7.0	6.6	1.1	1.3
MCTP2	1.0	8.5	1.3	10.7	8.5	8.2	1.0	1.3
CD44	2.9	17.4	2.1	13.5	6.0	6.6	0.9	0.7
ITGB8	1.6	12.1	1.4	12.2	7.8	8.7	0.9	0.9

## Discussion

The mechanism by which CypA restricts infection by some HIV-1 CA mutants is unknown. In this study we sought to test the hypothesis that a dominant-acting CypA-dependent restriction factor is responsible for CsA-dependent enhancement of these CA mutants. Assuming that the putative factor is differentially expressed in permissive and nonpermissive cells, we performed gene expression analysis on three permissive and three non-permissive cell lines. Based on our selection criteria, we focused on 26 candidate genes and individually knocked them down in HeLa cells using siRNAs. In order to thoroughly inhibit gene expression while minimizing cytotoxicity, we employed SMARTpool siRNAs and transfected cells using three different concentrations of siRNAs (8nM, 40 nM and 200 nM; Table 3 and data not shown). The concentrations of siRNAs used spanned the range of manufacturer-recommended concentrations. We expected a reversal of A92E phenotype when the putative restriction factor is depleted. Our data showed that depletion of at least nine genes resulted in 1.7–2.7-fold increase in A92E infectivity in the absence of CsA and 1.4–2.0-fold decrease in CsA dependence. Interestingly, of the nine genes, IFITM1 was demonstrated as a potent inhibitor of influenza A H1N1, dengue virus, West Nile virus and HIV-1 [37], [38]. Nevertheless, our results do not provide clear evidence for involvement of any of the 26 genes analyzed here in CsA enhancement of infection by HIV-1-A92E. It remains possible that low knock-down efficiency precluded identification of the putative gene. Alternatively, CypA-dependence could involve multiple gene products, thus single knock-downs may not reveal the restriction factor. Although this study focused on differences in expression levels, it is possible that single nucleotide polymorphisms could explain the phenotypic differences between cell types. Notably, previous studies have demonstrated that polymorphisms in the regulatory region of PPIA gene, which encodes CypA, influence susceptibility to HIV-1 infection or disease progression [39]–[41].

The CA mutants A92E and G94D were originally isolated as CypA-independent mutants by serial passage of wild-type HIV-1 in HeLa cells [15], [20]. These mutants are also dependent on CsA for their replication. Subsequently, it was established that the CsA-dependence of these mutants varies among cell lines [6], [7], [19], [24]. These mutants replicate efficiently in the presence of CsA in HeLa and H9 cells but not in Jurkat, HOS, TE671 and 293T cells. We identified another human T cell line, CEM, in which the A92E mutant requires CsA for infection. The cell-type dependence of the CsA-enhanced infection by these mutants is not understood. Evidence from heterokaryon analysis suggests that a dominant-acting CypA-dependent host factor inhibits A92E infection in HeLa cells [18]. Based on higher expression of CypA in non-permissive H9 cells compared to that in non-permissive Jurkat cells, it was suggested that differing CypA levels could explain the cell-type-dependent phenotype of the mutants [24]. Moreover, permissive TE671 cells overexpressing CypA were rendered non-permissive to infection by the mutants [19]. In contrast, overexpression of CypA in permissive 293T cells did not lead to enhancement of infection by the mutants upon CsA treatment [18]. Li et al. reported that the A92E mutant capsid undergoes more rapid uncoating in HeLa cells than in Jurkat cells, suggesting that CypA may inhibit this virus by destabilizing the capsid. [12]. Paradoxically, we have previously shown that CypA can stabilize wild type HIV-1 cores in vitro [42]. Moreover, a recent study demonstrated that CypA-CA interaction inhibited nuclear entry of A92E and increased the levels of pelletable CA protein in HeLa cells. The authors suggested that a dominant-acting restriction factor restricts the A92E mutant by stabilizing the viral capsid in a CypA-dependent manner [43]. Precedence for a CypA-dependent restriction factor exists in Old World monkey cells. In these cells, binding of CypA to the HIV-1 capsid leads to diminished HIV-1 infectivity by sensitizing the capsid to restriction by TRIM5α [44]–[46]. In human cells, CypA interaction with CA seemed to protect HIV-1 from a saturable restriction factor [47]. Initial studies in human cells suggested that huTRIM5α could restrict HIV-1 infection in a CypA-dependent manner [11], [48]. However, later studies demonstrated that endogenous human TRIM5α modestly inhibits HIV-1 and inhibition of CypA-CA interaction does not diminish its antiviral activity [46], [49], [50]. Collectively, the evidence is consistent with the presence of an unknown CypA-dependent restriction factor in cell types that are nonpermissive to CsA-dependent mutants. Interestingly, three recent studies have identified the human MxB protein as a CypA-dependent HIV-1 restriction factor that is induced by interferon [51]–[53].

One strong candidate for the CypA-dependent HIV-1 restriction factor is the host protein CPSF6. A C-terminally truncated CPSF6 (mCPSF6-358) protein, which localizes to the cytoplasm, was demonstrated to bind HIV-1 CA and inhibit viral nuclear entry [54]. The N74D substitution in CA renders the virus resistant to CPSF6-358 by inhibiting binding of CPSF6-358 to the capsid. Like wild-type HIV-1, A92E mutant is inhibited by CPSF6-358 [55]. Interestingly, the N74D/A92E double mutant is resistant to CPSF6-358 restriction, suggesting that N74D mutation rescues A92E presumably by preventing CPSF6 binding [55]. Moreover, infection by another CsA-dependent mutant T54A is rescued by addition of A105T, which is present in the CA binding site for CPSF6 and confers resistance to CPSF6-358 [56]. It was recently reported that CsA-dependent mutants are partially rescued by depletion of endogenous CPSF6 in HeLa cells [57]. However, expression levels of CPSF6 in permissive and non-permissive cell lines were similar in our study (Table 4), thus variations in its expression do not correlate with the cell-dependence of infection by the HIV-1-A92E mutant. Therefore, it remains unknown whether the cell-type-dependent phenotype of CsA-dependent mutants is accounted for by CPSF6.

**Table 4:** Analysis of known capsid-interacting proteins for CsA-dependent phenotype of A92E mutant.

Gene	Expression (log2)(RMA normalized)						Pearson correlationcoefficient
	HeLa	H9	CEM	Jurkat	HOS	293T	
PPIA	13.71	13.76	13.64	13.68	13.6	13.66	−0.46
PPIB	12.03	11.14	11.03	11.44	11.92	11.57	0.54
TNPO3	11.17	11.3	11.74	11.31	10.92	11.23	−0.11
CPSF6	11.15	11.27	11.38	11.46	11.12	11.39	−0.59
NUP358	11.04	10.88	10.84	10.86	10.42	11.3	0.16
NUP153	11.35	11.29	11.2	11.05	11.29	11.2	0.71
NUP98	11.67	11.25	11.17	11.32	11.33	11.17	0.88
ERK2	11.45	11.78	11.47	11.49	10.89	11.43	0.1
PIN1	9.04	9.72	9.38	9.71	8.99	9.65	−0.57
TRIM5	7.98	7.95	7.80	8.41	8.32	5.92	0.04

Expression of genes encoding known capsid-interacting proteins in permissive and non-permissive cell lines along with the Pearson correlation coefficient was obtained from Table S2.

The goal of this study was to identify a novel restriction factor in non-permissive cell types. We chose three permissive and three non-permissive cell types and performed human genome microarray analysis on these cell types. We grouped cell lines based on their permissivity to HIV-1-A92E infection and then compared the mean levels of gene expression in permissive versus non-permissive cell types to generate a ranked list of candidate genes. Our approach was mainly driven by the observation that the magnitude of CsA-dependence of A92E was ∼3-fold higher in HeLa cells than in other non-permissive cell types. Due to limited resources, we analyzed only 26 candidates from the first 100 genes whose expression in non-permissive cell types group was 3-fold or higher than in permissive cell types group. A limitation of this approach is that it ignores differences in CsA dependence within each group. An alternative approach involves correlating the fold-difference in CsA-dependent phenotype between cell types with fold-change in gene expression. To this end, we calculated a Pearson correlation coefficient for each gene to correlate the CsA-dependence of the mutant with gene expression (Table S3). This approach accounts for differences in CsA-dependence within each group. Of the 26 genes selected by the first approach, four genes (*SLC16A6, MCTP, ICAM1* and *TLL1*) had Pearson correlation coefficients in the range of 0.97–0.98 and were among the top-ranked 100 genes by the Pearson method. Moreover, none of the selected hits contain a CypA domain or associate with either CypA or CypA domain containing protein. Expression of genes encoding known HIV-1 capsid interaction partners [4], [14], [54], [58]–[61] were also analyzed (Table 4). Some studies have suggested that CypA levels dictate the CsA-dependent phenotype of the A92E mutant [19], [24]. However, the gene expression data showed a negative correlation between *CYPA* expression and CsA- dependence of the A92E mutant. Similarly, expression of CPSF6 was also negatively correlated with CsA-dependence of the mutant. *NUP153*, *NUP358*, *NUP98*, *ERK* and *CYPB* had positive but poor correlation with the CsA-dependent phenotype. Collectively, the alternative approach suggests that the known capsid-interacting proteins are unlikely to determine CsA-dependence of the A92E mutant.

In summary, although our limited study failed to identify the putative restriction factor, the approach using DNA microarray and the results thereof can be employed by other interested investigators for a more detailed analysis. The gene expression data may also be useful to investigators interested in other phenotypes that vary among the cell lines employed in this study.

## Supporting Information

Table S1 **Human gene array.** The table shows RMA normalized (log2 scale) values, mRNA accession numbers and gene symbols for all the genes analyzed by Human Gene 1.0 ST Array. The gene expression analysis was performed on six cell lines in triplicate.
(RAR)

Table S2 **Fold change in expression of genes in non-permissive vs permissive cells.** Mean expression values of each gene in permissive and non-permissive cell types were calculated. The mean value was used to calculate fold change in expression of each gene in permissive (CA1–9) vs non-permissive (CA10–18) cell types. Fold expression values with a (−) sign indicate higher expression in non-permissive cells, values with a (+) sign indicate higher expression in permissive cells. Columns also include annotation, uncorrected p-value, two p-values corrected by multiple testing correction (Bonferroni which is very stringent and then STepup also called Benjamini-Hochberg which controls the error in a different way but is less stringent).
(XLSX)

Table S3 **Correlation between CsA-dependent phenotype of the mutant with gene expression.** Pearson correlation coefficient for each gene was calculated to correlate the CsA-dependence of the mutant (calculated in Table 1) with gene expression. The list is sorted by the Pearson correlation coefficient value. Slope values were obtained by plotting CsA-dependent phenotype (X-axis) in all the cell lines against the gene expression values (Y-axis).
(XLSX)

## References

[1] Franke EK, Yuan HE, Luban J (1994) Specific incorporation of cyclophilin A into HIV-1 virions. Nature 372: 359–362. 7969494 10.1038/372359a0

[2] Gamble TR, Vajdos FF, Yoo S, Worthylake DK, Houseweart M, et al (1996) Crystal structure of human cyclophilin A bound to the amino-terminal domain of HIV-1 capsid. Cell 87: 1285–1294. 8980234 10.1016/s0092-8674(00)81823-1

[3] Gatanaga H, Das D, Suzuki Y, Yeh DD, Hussain KA, et al (2006) Altered HIV-1 Gag protein interactions with cyclophilin A (CypA) on the acquisition of H219Q and H219P substitutions in the CypA binding loop. J Biol Chem 281: 1241–1250. 16275650 10.1074/jbc.M505920200

[4] Luban J, Bossolt KL, Franke EK, Kalpana GV, Goff SP (1993) Human immunodeficiency virus type 1 Gag protein binds to cyclophilins A and B. Cell. 73: 1067–1078. 10.1016/0092-8674(93)90637-6 8513493

[5] Thali M, Bukovsky A, Kondo E, Rosenwirth B, Walsh CT, et al (1994) Functional association of cyclophilin A with HIV-1 virions. Nature 372: 363–365. 7969495 10.1038/372363a0

[6] Hatziioannou T, Perez-Caballero D, Cowan S, Bieniasz PD (2005) Cyclophilin interactions with incoming human immunodeficiency virus type 1 capsids with opposing effects on infectivity in human cells. J Virol 79: 176–183. 15596813 10.1128/JVI.79.1.176-183.2005 PMC538701

[7] Sokolskaja E, Sayah DM, Luban J (2004) Target cell cyclophilin A modulates human immunodeficiency virus type 1 infectivity. J Virol 78: 12800–12808. 15542632 10.1128/JVI.78.23.12800-12808.2004 PMC524981

[8] Handschumacher RE, Harding MW, Rice J, Drugge RJ, Speicher DW (1984) Cyclophilin: a specific cytosolic binding protein for cyclosporin A. Science. 226: 544–547. 10.1126/science.6238408 6238408

[9] Ikeda Y, Ylinen LM, Kahar-Bador M, Towers GJ (2004) Influence of gag on human immunodeficiency virus type 1 species-specific tropism. J Virol 78: 11816–11822. 15479823 10.1128/JVI.78.21.11816-11822.2004 PMC523279

[10] Kootstra NA, Munk C, Tonnu N, Landau NR, Verma IM (2003) Abrogation of postentry restriction of HIV-1-based lentiviral vector transduction in simian cells. Proc Natl Acad Sci U S A 100: 1298–1303. 12547912 10.1073/pnas.0337541100 PMC298767

[11] Towers GJ, Hatziioannou T, Cowan S, Goff SP, Luban J, et al (2003) Cyclophilin A modulates the sensitivity of HIV-1 to host restriction factors. Nat Med 9: 1138–1143. 12897779 10.1038/nm910

[12] Li Y, Kar AK, Sodroski J (2009) Target cell type-dependent modulation of human immunodeficiency virus type 1 capsid disassembly by cyclophilin A. J Virol. 83: 10951–10962. 10.1128/JVI.00682-09 PMC2772774 19656870

[13] Qi M, Yang R, Aiken C (2008) Cyclophilin A-dependent restriction of human immunodeficiency virus type 1 capsid mutants for infection of nondividing cells. J Virol 82: 12001–12008. 18829762 10.1128/JVI.01518-08 PMC2593355

[14] Schaller T, Ocwieja KE, Rasaiyaah J, Price AJ, Brady TL, et al (2011) HIV-1 capsid-cyclophilin interactions determine nuclear import pathway, integration targeting and replication efficiency. PLoS Pathog 7: e1002439. 22174692 10.1371/journal.ppat.1002439 PMC3234246

[15] Aberham C, Weber S, Phares W (1996) Spontaneous mutations in the human immunodeficiency virus type 1 gag gene that affect viral replication in the presence of cyclosporins. J Virol 70: 3536–3544. 8648687 10.1128/jvi.70.6.3536-3544.1996 PMC190228

[16] Rosenwirth B, Billich A, Datema R, Donatsch P, Hammerschmid F, et al (1994) Inhibition of human immunodeficiency virus type 1 replication by SDZ NIM 811, a nonimmunosuppressive cyclosporine analog. Antimicrob Agents Chemother 38: 1763–1772. 7527198 10.1128/aac.38.8.1763 PMC284634

[17] Wainberg MA, Dascal A, Blain N, Fitz-Gibbon L, Boulerice F, et al (1988) The effect of cyclosporine A on infection of susceptible cells by human immunodeficiency virus type 1. Blood 72: 1904–1910. 2904290

[18] Song C, Aiken C (2007) Analysis of human cell heterokaryons demonstrates that target cell restriction of cyclosporine-resistant human immunodeficiency virus type 1 mutants is genetically dominant. J Virol 81: 11946–11956. 17715216 10.1128/JVI.00620-07 PMC2168785

[19] Ylinen LM, Schaller T, Price A, Fletcher AJ, Noursadeghi M, et al (2009) Cyclophilin A levels dictate infection efficiency of human immunodeficiency virus type 1 capsid escape mutants A92E and G94D. J Virol 83: 2044–2047. 19073742 10.1128/JVI.01876-08 PMC2643744

[20] Braaten D, Franke EK, Luban J (1996) Cyclophilin A is required for an early step in the life cycle of human immunodeficiency virus type 1 before the initiation of reverse transcription. J Virol 70: 3551–3560. 8648689 10.1128/jvi.70.6.3551-3560.1996 PMC190230

[21] Schneidewind A, Brockman MA, Yang R, Adam RI, Li B, et al (2007) Escape from the dominant HLA-B27-restricted cytotoxic T-lymphocyte response in Gag is associated with a dramatic reduction in human immunodeficiency virus type 1 replication. J Virol 81: 12382–12393. 17804494 10.1128/JVI.01543-07 PMC2169010

[22] Takemura T, Kawamata M, Urabe M, Murakami T (2013) Cyclophilin A-dependent restriction to capsid N121K mutant human immunodeficiency virus type 1 in a broad range of cell lines. J Virol 87: 4086–4090. 23325683 10.1128/JVI.01319-12 PMC3624240

[23] Yang R, Aiken C (2007) A mutation in alpha helix 3 of CA renders human immunodeficiency virus type 1 cyclosporin A resistant and dependent: rescue by a second-site substitution in a distal region of CA. J Virol 81: 3749–3756. 17267487 10.1128/JVI.02634-06 PMC1866112

[24] Yin L, Braaten D, Luban J (1998) Human immunodeficiency virus type 1 replication is modulated by host cyclophilin A expression levels. J Virol 72: 6430–6436. 9658084 10.1128/jvi.72.8.6430-6436.1998 PMC109799

[25] Gallay P, Hope T, Chin D, Trono D (1997) HIV-1 infection of nondividing cells through the recognition of integrase by the importin/karyopherin pathway. Proc Natl Acad Sci U S A 94: 9825–9830. 9275210 10.1073/pnas.94.18.9825 PMC23276

[26] He J, Chen Y, Farzan M, Choe H, Ohagen A, et al (1997) CCR3 and CCR5 are co-receptors for HIV-1 infection of microglia. Nature 385: 645–649. 9024664 10.1038/385645a0

[27] Yee JK, Friedmann T, Burns JC (1994) Generation of high-titer pseudotyped retroviral vectors with very broad host range. Methods Cell Biol 43 Pt A: 99–112. 10.1016/s0091-679x(08)60600-7 7823872

[28] Deng H, Liu R, Ellmeier W, Choe S, Unutmaz D, et al (1996) Identification of a major co-receptor for primary isolates of HIV-1. Nature 381: 661–666. 8649511 10.1038/381661a0

[29] Foley GE, Lazarus H, Farber S, Uzman BG, Boone BA, et al (1965) Continuous Culture of Human Lymphoblasts from Peripheral Blood of a Child with Acute Leukemia. Cancer 18: 522–529. 14278051 10.1002/1097-0142(196504)18:4<522::aid-cncr2820180418>3.0.co;2-j

[30] Landau NR, Littman DR (1992) Packaging system for rapid production of murine leukemia virus vectors with variable tropism. J Virol 66: 5110–5113. 1321291 10.1128/jvi.66.8.5110-5113.1992 PMC241381

[31] Popovic M, Read-Connole E, Gallo RC (1984) T4 positive human neoplastic cell lines susceptible to and permissive for HTLV-III. Lancet 2: 1472–1473. 10.1016/s0140-6736(84)91666-0 6151082

[32] Weiss A, Wiskocil RL, Stobo JD (1984) The role of T3 surface molecules in the activation of human T cells: a two-stimulus requirement for IL 2 production reflects events occurring at a pre-translational level. J Immunol 133: 123–128. 6327821

[33] Kiernan RE, Ono A, Englund G, Freed EO (1998) Role of matrix in an early postentry step in the human immunodeficiency virus type 1 life cycle. J Virol 72: 4116–4126. 9557701 10.1128/jvi.72.5.4116-4126.1998 PMC109641

[34] Naldini L, Blomer U, Gage FH, Trono D, Verma IM (1996) Efficient transfer, integration, and sustained long-term expression of the transgene in adult rat brains injected with a lentiviral vector. Proc Natl Acad Sci U S A 93: 11382–11388. 8876144 10.1073/pnas.93.21.11382 PMC38066

[35] Chen C, Okayama H (1987) High-efficiency transformation of mammalian cells by plasmid DNA. Mol Cell Biol 7: 2745–2752. 3670292 10.1128/mcb.7.8.2745-2752.1987 PMC367891

[36] Wehrly K, Chesebro B (1997) p24 antigen capture assay for quantification of human immunodeficiency virus using readily available inexpensive reagents. Methods 12: 288–293. 9245608 10.1006/meth.1997.0481

[37] Brass AL, Huang IC, Benita Y, John SP, Krishnan MN, et al (2009) The IFITM proteins mediate cellular resistance to influenza A H1N1 virus, West Nile virus, and dengue virus. Cell 139: 1243–1254. 20064371 10.1016/j.cell.2009.12.017 PMC2824905

[38] Lu J, Pan Q, Rong L, He W, Liu SL, et al (2011) The IFITM proteins inhibit HIV-1 infection. J Virol 85: 2126–2137. 21177806 10.1128/JVI.01531-10 PMC3067758

[39] An P, Wang LH, Hutcheson-Dilks H, Nelson G, Donfield S, et al (2007) Regulatory polymorphisms in the cyclophilin A gene, PPIA, accelerate progression to AIDS. PLoS Pathog 3: e88. 17590083 10.1371/journal.ppat.0030088 PMC1894826

[40] Bleiber G, May M, Martinez R, Meylan P, Ott J, et al (2005) Use of a combined ex vivo/in vivo population approach for screening of human genes involved in the human immunodeficiency virus type 1 life cycle for variants influencing disease progression. J Virol 79: 12674–12680. 16188970 10.1128/JVI.79.20.12674-12680.2005 PMC1235818

[41] Rits MA, van Dort KA, Kootstra NA (2008) Polymorphisms in the regulatory region of the Cyclophilin A gene influence the susceptibility for HIV-1 infection. PLoS One 3: e3975. 19092998 10.1371/journal.pone.0003975 PMC2599883

[42] Shah VB, Shi J, Hout DR, Oztop I, Krishnan L, et al (2013) The host proteins transportin SR2/TNPO3 and cyclophilin A exert opposing effects on HIV-1 uncoating. J Virol 87: 422–432. 23097435 10.1128/JVI.07177-11 PMC3536424

[43] De Iaco A, Luban J (2014) Cyclophilin A promotes HIV-1 reverse transcription but its effect on transduction correlates best with its effect on nuclear entry of viral cDNA. Retrovirology 11: 11. 24479545 10.1186/1742-4690-11-11 PMC3916700

[44] Berthoux L, Sebastian S, Sokolskaja E, Luban J (2005) Cyclophilin A is required for TRIM5{alpha}-mediated resistance to HIV-1 in Old World monkey cells. Proc Natl Acad Sci U S A 102: 14849–14853. 16203999 10.1073/pnas.0505659102 PMC1239943

[45] Keckesova Z, Ylinen LM, Towers GJ (2006) Cyclophilin A renders human immunodeficiency virus type 1 sensitive to Old World monkey but not human TRIM5 alpha antiviral activity. J Virol 80: 4683–4690. 16641261 10.1128/JVI.80.10.4683-4690.2006 PMC1472055

[46] Stremlau M, Perron M, Lee M, Li Y, Song B, et al (2006) Specific recognition and accelerated uncoating of retroviral capsids by the TRIM5alpha restriction factor. Proc Natl Acad Sci U S A 103: 5514–5519. 16540544 10.1073/pnas.0509996103 PMC1459386

[47] Towers G, Collins M, Takeuchi Y (2002) Abrogation of Ref1 retrovirus restriction in human cells. J Virol 76: 2548–2550. 11836433 10.1128/jvi.76.5.2548-2550.2002 PMC153820

[48] Sayah DM, Luban J (2004) Selection for loss of Ref1 activity in human cells releases human immunodeficiency virus type 1 from cyclophilin A dependence during infection. J Virol 78: 12066–12070. 15479848 10.1128/JVI.78.21.12066-12070.2004 PMC523284

[49] Sokolskaja E, Berthoux L, Luban J (2006) Cyclophilin A and TRIM5alpha independently regulate human immunodeficiency virus type 1 infectivity in human cells. J Virol 80: 2855–2862. 16501094 10.1128/JVI.80.6.2855-2862.2006 PMC1395419

[50] Stremlau M, Song B, Javanbakht H, Perron M, Sodroski J (2006) Cyclophilin A: an auxiliary but not necessary cofactor for TRIM5alpha restriction of HIV-1. Virology 351: 112–120. 16643975 10.1016/j.virol.2006.03.015

[51] Goujon C, Moncorge O, Bauby H, Doyle T, Ward CC, et al (2013) Human MX2 is an interferon-induced post-entry inhibitor of HIV-1 infection. Nature 502: 559–562. 24048477 10.1038/nature12542 PMC3808269

[52] Kane M, Yadav SS, Bitzegeio J, Kutluay SB, Zang T, et al (2013) MX2 is an interferon-induced inhibitor of HIV-1 infection. Nature 502: 563–566. 24121441 10.1038/nature12653 PMC3912734

[53] Liu Z, Pan Q, Ding S, Qian J, Xu F, et al (2013) The interferon-inducible MxB protein inhibits HIV-1 infection. Cell Host Microbe 14: 398–410. 24055605 10.1016/j.chom.2013.08.015

[54] Lee K, Ambrose Z, Martin TD, Oztop I, Mulky A, et al (2010) Flexible use of nuclear import pathways by HIV-1. Cell Host Microbe 7: 221–233. 20227665 10.1016/j.chom.2010.02.007 PMC2841689

[55] De Iaco A, Santoni F, Vannier A, Guipponi M, Antonarakis S, et al (2013) TNPO3 protects HIV-1 replication from CPSF6-mediated capsid stabilization in the host cell cytoplasm. Retrovirology 10: 20. 23414560 10.1186/1742-4690-10-20 PMC3599327

[56] Price AJ, Fletcher AJ, Schaller T, Elliott T, Lee K, et al (2012) CPSF6 Defines a Conserved Capsid Interface that Modulates HIV-1 Replication. PLoS Pathog 8: e1002896. 22956906 10.1371/journal.ppat.1002896 PMC3431306

[57] Henning MS, Dubose BN, Burse MJ, Aiken C, Yamashita M (2014) In Vivo Functions of CPSF6 for HIV-1 as Revealed by HIV-1 Capsid Evolution in HLA-B27-Positive Subjects. PLoS Pathog 10: e1003868. 24415937 10.1371/journal.ppat.1003868 PMC3887095

[58] Di Nunzio F, Fricke T, Miccio A, Valle-Casuso JC, Perez P, et al (2013) Nup153 and Nup98 bind the HIV-1 core and contribute to the early steps of HIV-1 replication. Virology 440: 8–18. 23523133 10.1016/j.virol.2013.02.008 PMC3860269

[59] Gupta P, Singhal PK, Rajendrakumar P, Padwad Y, Tendulkar AV, et al (2011) Mechanism of host cell MAPK/ERK-2 incorporation into lentivirus particles: characterization of the interaction between MAPK/ERK-2 and proline-rich-domain containing capsid region of structural protein Gag. J Mol Biol 410: 681–697. 21762808 10.1016/j.jmb.2011.03.022

[60] Misumi S, Inoue M, Dochi T, Kishimoto N, Hasegawa N, et al (2010) Uncoating of human immunodeficiency virus type 1 requires prolyl isomerase Pin1. J Biol Chem 285: 25185–25195. 20529865 10.1074/jbc.M110.114256 PMC2919081

[61] Zhou L, Sokolskaja E, Jolly C, James W, Cowley SA, et al (2011) Transportin 3 promotes a nuclear maturation step required for efficient HIV-1 integration. PLoS Pathog 7: e1002194. 21901095 10.1371/journal.ppat.1002194 PMC3161976

